# The Great Recession and Drinking Outcomes: Protective Effects of Politically Oriented Coping

**DOI:** 10.1155/2014/646451

**Published:** 2014-09-14

**Authors:** Judith A. Richman, Robyn Lewis Brown, Kathleen M. Rospenda

**Affiliations:** ^1^Department of Psychiatry, University of Illinois at Chicago, 1601 W. Taylor Street, Chicago, IL 60612, USA; ^2^Department of Sociology, DePaul University, 990 W. Fullerton Avenue, Suite 1100, Chicago, IL 60614, USA

## Abstract

Research derived from the stress paradigm suggests that certain types of coping (e.g., problem-focused coping instead of behavioral disengagement) are protective against problem-related drinking to deal with social stressors. Going beyond the typical focus in the coping literature, we hypothesize that stressors engendered by macrolevel social forces may require coping actions within the political realm in contrast to modes of coping focused outside of the political realm. A United States sample of 663 respondents completed a mail survey in 2010, including measures of stressful consequences of the Great Recession, drinking patterns and problems, modes of coping encompassed in the Brief COPE instrument, and politically oriented coping. Structural equation modeling examined whether modes of coping mediated the links between stressors and drinking outcomes. A substantial portion of the associations between stressors and drinking was explained by modes of coping. Politically oriented coping was protective against problem drinking for both genders. Future studies should further explore politically oriented coping in addition to modes of coping outside of the political realm when studying the relationships between macrolevel social stressors and deleterious drinking outcomes.

## 1. Introduction

Social scientists who explore factors mediating and moderating the relationships between social stressors and mental health, including drinking outcomes, have highlighted modes of coping [1, 2]. These studies have explored behaviors which protect people from being psychologically harmed [3] and cognitive appraisals which influence behaviors such as problem-focused coping [4] or using alcohol to self-medicate distress [5]. However, studies have not considered the characteristics of the stressful situation itself that may make certain coping strategies more or less effective [6]. In particular, psychiatric epidemiologic studies have tended to emphasize microlevel stressors (e.g., stressors in individuals' role domains) and, until recently, have ignored the linkages between macrolevel social forces and the daily stressors in people's lives [7–9]. However, macrolevel social conditions can affect the magnitude of stressors experienced in people's lives and the extent to which they experience “cumulative adversity” [10]. This paper focuses on coping with the fallout from one type of macrolevel social stressor: the recent Great Recession. This economic downturn constituted the most severe economic crisis in the United States since the Great Depression [11] and had persisting economic effects (e.g., job loss, less desirable working conditions, loss of home, loss of retirement savings, lack of health care access, and social isolation) which have been linked with deleterious drinking outcomes [12].

A key issue involving the effectiveness of alternative modes of coping with stressors derived from macrolevel social forces and protecting against deleterious drinking outcomes is the question of whether individual modes of coping outside of the political realm emphasized in the overall coping literature are most efficacious. Or, alternatively, do stressors engendered by macrolevel social forces require unique forms of coping, that encompass the political realm? With respect to coping strategies employed in the wake of the Great Recession, politically oriented coping strategies might be of particular relevance due to the impact of governmental decision-making on the state of the economy. Politically oriented coping strategies might include political activism oriented to altering economic policies or support for campaigns by politicians offering solutions to economically based hardships. Scholarly work on the Occupy Wall Street movement, for example, documents involvement by individuals sharing their own economic struggles [13] and collectively gathering to protest their precarious economic situation and uncertain economic future [14]. Earlier work also suggests the salience of activities oriented toward changing politically based social realities such as through the act of voting [15] or by collectively challenging community-level decisions such as school closings [16]. By contrast, the traditional coping literature has emphasized individual, nonpolitical modes of coping, such as emotional acceptance of the stressful situation, blaming one's self for the situation, or taking individual actions such as looking for a job if unemployed [17].

While sociologists have recently accorded greater attention to the prevalence and alcohol-related consequences of macrolevel stressors, they have yet to address the extent to which politically oriented modes of coping may be the most efficacious ways to address problems stemming from macrolevel social forces or events. Prior to the more recent focus on macrolevel stressors, Kaplan and Liu [18] embraced the idea of collective coping as one means for individuals who maintained stigmatized personal identities to challenge and transform conventional socionormative systems through participation in social movements. Subsequently, Thoits [19] more explicitly addressed collective coping within the context of the stress paradigm involving acute and chronic stressors not limited to the specific area of stigmatized statuses. She argued that individuals who find themselves in problematic situations can deliberately work to transform the meaning of their experiences and they can additionally use these experiences “as a basis for helping or effecting changes in the lives of others” [19, page 314]. She proposed the concept of “transformatory coping” to include engagement in collective activist activities with others who share similar problems. For example, parents of autistic children have lobbied governments for social services perceived to aid in their children's development. In sum, (1) collective coping tactics (such as politically oriented coping) represent an unmeasured dimension of coping behaviors beyond that represented in the coping literature to date and (2) collective coping tactics may demonstrate a stronger association between stressors, particularly those stemming from macrolevel social forces, and deleterious drinking outcomes compared to the use of modes of coping previously emphasized in the literature on coping.

The present study extends previous work by empirically addressing the extent to which politically oriented coping activities engaged in as a response to a macrolevel social stressor, the Great Recession, are protective against alcohol-related outcomes compared with coping strategies focused outside of the political realm. We hypothesize that politically oriented coping will be more protective against economic stressors linked with the Great Recession than nonpolitical modes of coping and will uniquely account for some portion of the associations between economy-related stressors and drinking outcomes.

Further, we also examine whether politically oriented coping and coping outside of the political realm are more protective for men versus women in the face of macrolevel engendered stressors such as those involving the economic fallout from the Great Recession. There is consistent evidence that women are more likely to use support-based coping strategies (e.g., seeking support from others such as partner family and friends) in response to stress in contrast to men and some indication that avoidant coping techniques are associated with greater alcohol consumption among men but not women [1, 20–22]. (In contrast, parity by gender in the use of individual active coping strategies and their significance for mental health outcomes is generally reported [1, 21].) However, whether there is a corresponding propensity for men and women to differ in the use of politically oriented coping to offset the alcohol-related effects of economy-related stressors is less certain. Earlier research tended to argue that women were less politically interested, informed, and efficacious compared to men [23]. More recent work has shown that women and men differ in particular modes of participation; women are more likely to vote and engage in individual political actions such as signing petitions or donating money, whereas men are more likely to be engaged in collective forms of action such as group protest activities [24]. Thus, we hypothesize that there will be no overall differences in the extent to which women and men manifest politically oriented coping or in the effect that politically oriented coping has on drinking outcomes. Following a transactional model of stress [4, 25], we model coping as a mediator of the relationship between the stressor (i.e., stressful consequences of the Great Recession) and the stress response (i.e., drinking outcomes).

## 2. Methods 

### 2.1. Study Procedures and Sample

Data were derived from a study conducted in the United States between June, 2010, and January, 2011, that was undertaken in order to understand life change consequences of the major downturn in the economy known as the Great Recession. Respondents were selected by a random digit dial (RDD) phone survey of the continental United States, and those who consented to participate in the study were mailed questionnaires. The phone screener was conducted using computerized assisted telephone interview (CATI) software. Eligibility criteria involved being aged 18 years or older and English-speaking. Eligible respondents were selected from the households using the Troldahl-Carter-Bryant method of respondent selection which involves the means to randomly select a respondent from all eligible household members [26]. Respondents were told during the phone screen that a $50 American Express gift card would be sent to the eligible respondent if he or she completed the questionnaire. Respondents were mailed an initial survey, a postcard reminder to nonresponders, and a second questionnaire if they still had not responded.

**Figure 1:**
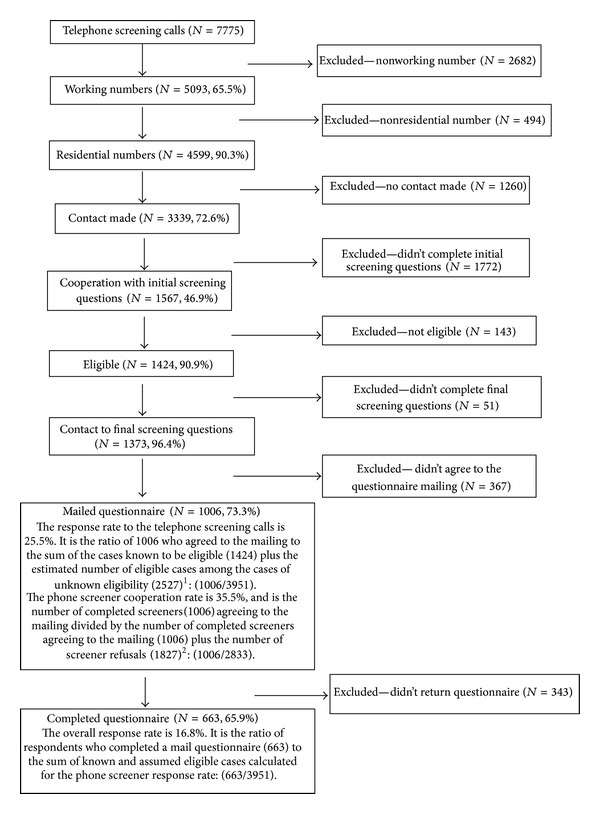
A summary of sample Ns and response rates at each decision point. ^1^There were 2416 cases for which a screening questionnaire could not be administered (contact to screener minus cooperation to screener plus answering machines). We assumed that 90.9% of these cases would have been eligible. In another 616 cases, the phone rang continuously at each contact attempt and was never answered. We assumed that 65.5% of those were working numbers, 90.3% were household numbers, and 90.9% were eligible. Consequently, the total number of cases with assumed eligibility is estimated as 90.9% of 2416 (2196) plus 53.8% of 616 (331 cases) or 2527. ^2^Screener refusals include actual refusals of eligible respondents plus a proportion of refusals of households whose eligibility is unknown. The total number of phone screener refusals is those who refused after screening, 367 (including 353 who refused the interview; 11 who cited the “Do not call” registry as a reason for refusal after being screened; and 3 who cited privacy manager as a reason for refusal after being screened), plus 90.9% of the 1,606 who refused prior to screening (including 1483 who refused before completing the screener; 37 who cited the “Do not call” registry as reason refusal; and 86 who cited privacy manager as a reason for refusal), for a total of 1827 phone screener refusals.

A total of 7,775 telephone screening calls were initially made.  Figure 1 encompasses a flow chart characterizing each stage in the data collection process through the completion of the questionnaires. 65.9% (*n* = 663) of the respondents completing the screening calls returned the questionnaire. The telephone screening cooperation rate and the mail survey response rate were each calculated using the conservative AAPOR response rate formula 3 [27]. The overall survey response rate is the product of the phone screening cooperation rate (35.5%) and the mail questionnaire return response rate (65.9%) or 16.8%. We acknowledge that this response rate is less than ideal and further address this issue in the discussion of study limitations and note other indicators of the representativeness of the final sample.

The final sample obtained was weighted in two ways. Selection weights were calculated for each of the cases to weight for the different probability of selection for each case. Poststratification weights were calculated for the dataset to ensure that the distribution of sample cases on important demographic variables (age, race/ethnicity, and gender) conformed to the distribution of these variables in Census Bureau's 2008 United States Population Estimates. It should be noted that estimates of alcohol consumption for the present sample did appear to conform to national estimates preweighting. For example, the average number of drinks consumed in the past month on days when one drank for the present sample is 2.16, versus the estimated average of 2.10 reported by the Centers for Disease Control and Prevention's Behavioral Risk Factor Surveillance System for 2010 [28] (by gender, the averages are 2.35 for men and 1.89 for women in the present sample versus 2.43 for men and 1.81 for women in the CDC estimates; by race/ethnicity, the averages are 2.16 for non-Hispanic Whites, 2.08 for Asians, 2.12 for African Americans, and 2.72 for Hispanics in the present sample versus 2.26 for non-Hispanic Whites, 2.41 for Asians, 2.19 for African Americans, and 2.68 for Hispanics in the CDC data).

It should additionally be noted that the respondents included in this sample reported an overall higher level of education than the general population based on 2008 Census estimates. Analysis of variance revealed no significant variation by education in either the outcomes or support coping, avoidant coping, or politically oriented coping. Respondents with less than a high school degree are found to be marginally less likely (*P* < 0.10) to use active coping strategies compared to those with a college degree or postcollege training. Given this discrepancy as well as the fact that education is generally protective against problem drinking, education is included as a control variable in all of the analyses presented.

### 2.2. Measures

Summary statistics for all study variables are found in Table 1. Two outcomes are considered: past-month drinking patterns and problematic drinking patterns. Predictor variables are economy-related stressors, coping strategies enacted outside of the political realm (i.e., active coping, support coping, and avoidant coping), politically oriented coping, and gender. The sociodemographic characteristics of age, education, and race/ethnicity are controlled in all analyses.

#### 2.2.1. Past-Month Drinking Patterns

To assess past-month drinking patterns, we use the Quantity-Frequency-Variability Index (QFV) developed by Cahalan et al. [29]. Frequency of drinking is measured as a count of the days on which alcohol was consumed in the past 30 days, and quantity of drinking is measured as the average number of drinks consumed on those days. Variability is calculated by the greatest number of drinks consumed on any one day in the past 30 days. Scores are calculated by multiplying responses to the quantity, frequency, and variability questions (*α* = 0.87). As with the current data, this index tends to approximate a continuous scale, and ample evidence supports its use as such (see Fitzgerald and Mulford [30] for a review).

#### 2.2.2. Problem Drinking

Our measure of problematic drinking is the 10-item BMAST (*α* = 0.74) [31], which is a count measure of difficulties related to alcohol use over the past year. Respondents were asked to indicate “yes” or “no” in response to 10 items such as having an accident, losing a close friend, spouse, or loved one, being hospitalized, having trouble at work, and soliciting professional help because of one's drinking. The BMAST is one of the most widely used tools for assessing alcohol dependence and problems [32]. It correlates strongly with the full-length MAST and evidence of its reliability and validity is widely available [31, 32]. Moreover, we are not using the bMAST as a diagnostic tool for depicting “problem” versus “nonproblem” drinking, but as it is intended, as suggestive of different degrees of problematic drinking.

#### 2.2.3. Economy-Related Stressors

The measure of economy-related stressors is the Life Change Consequences of the Great Recession (LCCGR) instrument [12]. This construct was developed on the basis of qualitative analyses of transcripts derived from focus groups involving both genders and diverse racial/ethnic groups. The final 39-item instrument was developed on the basis of confirmatory factor analysis. The alpha coefficients were 0.91 for women and 0.94 for men. The LCCGR has been shown to predict both drinking outcomes and psychological distress [33]. The items included in this inventory are published by Richman et al. [12]. The items fall into seven categories: home ownership problems, such as difficulties in mortgage payments, difficulties in paying property taxes, or a drop in credit rating; undesirable living situation, including having to live in a less desired location to save money or having gas and electricity or heat shut off due to an inability to pay bills; problematic employment situation, including a pay-cut, furlough days, and increased feelings of competition with fellow employees; unemployment or underemployment; inadequate health insurance, including lack of medical or dental coverage, decreased quality of coverage, and inability to obtain coverage; social role constraints, such as dissolution of spouse/partner relationship, decreased social life, and increased social isolation due to finances; and inadequate sick time, including inadequate sick days and having to work despite poor health. Consistent with common practice, each score for this measure is a straight count of the number of stressors reported.

#### 2.2.4. Modes of Coping outside of the Political Realm

Three dimensions of nonpolitical modes of coping are assessed:* active coping*,* support coping*, and* avoidant coping*. These measures of coping are derived from subscales of the Brief COPE instrument [17] and have previously been validated as stand-alone indices in community samples [34–36]. Participants in the present study were asked whether they have used these coping strategies in response to the economic recession. Confirmatory factor analysis of the present data supports the inclusion of these items as three separate coping indices, consistent with prior research. Active coping (*α* = 0.84) includes eight items measuring acceptance, positive reframing, and planning and taking action in response to the economic recession. Support coping (*α* = 0.81) includes four items measuring use of emotional and instrumental support (i.e., receiving emotional support and getting advice and help from other people). Avoidant coping (*α* = 0.75) includes 10 items measuring self-distraction, behavioral disengagement, self-denial, blame, and a tendency to vent about or make fun of the situation. All items were rated on a four-point Likert-type scale ranging from 0 (I did not do this at all) to 3 (I did this a lot).

#### 2.2.5. Politically Oriented Coping

The measure of politically oriented coping is assessed by a four-item instrument (*α* = 0.79) drawn from the summed responses (i.e., not at all, a little, some, quite a bit, and a lot) to four statements asking how often respondents, in response to the economic recession, have been (1) “engaging in political activities such as signing petitions, leading or participating in rallies or marches, or writing to political representatives;” (2) “organizing with others to challenge politicians currently in office;” (3) “voting in elections to support politicians who share your political beliefs;” and (4) “participating in groups trying to influence the policies of the government at the local, state, or national level.” The questions used to construct this index were derived from analyses of focus group transcripts (see [12] for details about these focus groups). Confirmatory factor analysis reveals that these items load on a single factor, supporting their inclusion as one index.

#### 2.2.6. Gender 

It is coded 1 for females and 0 for males.

#### 2.2.7. SociodemographicControl Variables

*Age* is employed as a continuous measure in years.* Education* is a categorical variable based on the educational attainment categories of (1) less than high school (*n* = 45); (2) high school graduate (*n* = 350); (3) college graduate (*n* = 110); and (4) postcollege training (*n* = 150).* Race/ethnicity* is a dummy variable including non-Hispanic Whites (*n* = 436), African Americans (*n* = 80), Hispanics (*n* = 91), Asians (*n* = 29), and individuals who identify as an “other” race/ethnicity (*n* = 17). In all analyses, non-Hispanic Whites serve as the reference category.

### 2.3. Data Analysis

After examining bivariate correlations in order to assess the basic patterns of association among key study variables, we performed structural equation modeling (SEM) using Mplus software [37] to examine the predictive significance of economy-related stressors for coping outside of the political realm and politically oriented coping tactics and the two drinking outcomes considered (i.e., past-month drinking and problematic drinking), net of the sociodemographic control variables. We considered the potential for nonpolitical coping and politically oriented coping tactics to mediate the associations between economy-related stressors and each of the outcomes assessed in two models. The first model tested for associations between economic stressors and the drinking-related outcomes. The second model adds nonpolitical and politically oriented coping tactics to test the full mediation model. This latter model assesses all of the direct and indirect paths between economic stressors and the drinking outcomes considered through the nonpolitical and politically oriented coping tactics investigated. We formally tested for mediation using the procedures described by Muthen and Muthen [37] for Mplus software, which apply the tests described by MacKinnon et al. [38].

Finally, because the associations between social stress and drinking are found to vary by gender [33, 39], we examined whether any observed mediating effects of the coping strategies investigated vary by gender. For these tests, separate equations include the interaction term for gender by each coping strategy in the path models linking coping with drinking outcomes.

## 3. Results

Table 2 presents the intercorrelations of major study variables. It is noteworthy that stressors related to the economy are associated with each of the alcohol-related outcomes and all of the coping resources considered: economy-related stressors are associated with more alcohol consumption and problematic drinking, as well as higher levels of active coping, support coping, avoidant coping, and politically oriented coping. It is also noteworthy that only two of the coping strategies assessed are associated with the drinking outcomes. Avoidant coping and politically oriented coping are associated with both alcohol consumption and problematic drinking, but in opposite directions. That is, greater avoidant coping is associated with greater alcohol consumption and problematic drinking, whereas greater politically oriented coping is associated with less alcohol consumption and problematic drinking. The lack of correlation between active coping and support coping and each of the drinking outcomes, respectively, provides some indication that not all of the coping strategies may be useful in understanding the associations between economic stressors and drinking-related outcomes.

Additionally, the possibility that coping resources may vary by gender is not strongly supported by the pattern of correlations reported, with the exception that women reported significantly more emotional support than men. However, and consistent with previous research [33], women are found to drink less and less problematically.

The hypothesized associations between economy-related stressors, coping strategies, and the alcohol-related outcomes are further elaborated upon in the structural equation model.

**Figure 2:**
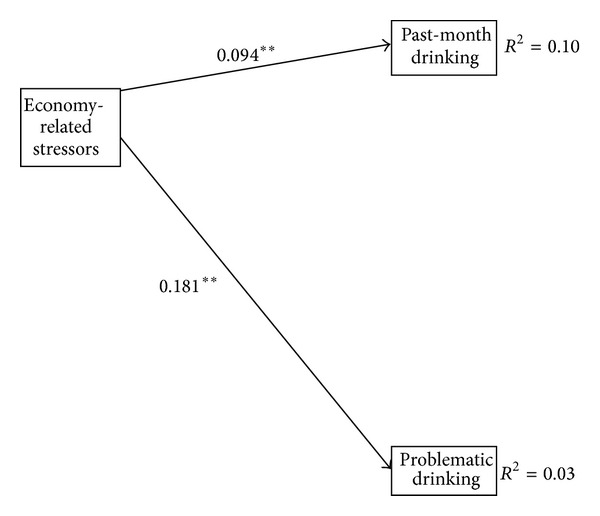
Structural equation model relating economy-related stressors to alcohol-related outcomes. Notes: standardized parameter estimates are reported. Model controls for gender, age, education, and race/ethnicity. ∗significant at 0.05; ∗∗significant at 0.01; ∗∗∗significant at 0.001.

Estimation of the first model (Figure 2), including only economic stressors, the drinking-related outcomes, and sociodemographic controls, produces a just identified model and, as such, meaningful fit statistics are not provided. The standardized path coefficients demonstrate that economic stressors and each of the drinking-related outcomes considered are significantly and positively related. Net of the sociodemographic controls, greater economic strain is associated with greater alcohol consumption over the past month (*β* = 0.094, s.e. = 0.004, and *P* < 0.01) and a higher incidence of problematic drinking over the past year (*β* = 0.181, s.e. = 0.008, and *P* < 0.01).

**Figure 3:**
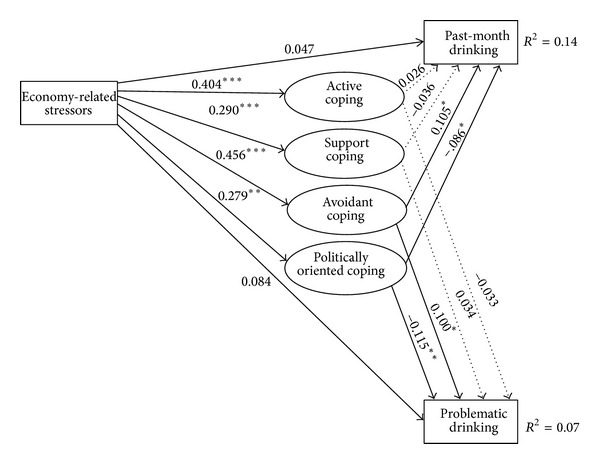
Structural equation model relating economy-related stressors to coping strategies and alcohol-related outcomes.

SEM analysis testing the second model, of the hypothesized associations between economy-related stressors, coping strategies, and the alcohol-related outcomes considered, is presented as Figure 3. In Figure 3, solid lines indicate effects that are statistically significant, and dashed lines indicate effects that are not statistically significant. The model fit criteria provided by Hu and Bentler [40] (CFI > 0.95; RMSEA < 0.06; SRMR < 0.08) are used to assess the measurement model. Based on these criteria, there is consistent evidence of good fit for this model (CFI = 0.982; RMSEA = 0.046; SRMR = 0.026).

This model demonstrates that economic stress is associated with higher levels of each of the coping resources considered. That is, greater economic strain appears to predict a greater propensity to use both maladaptive coping tactics (i.e., avoidant coping) and adaptive coping tactics (i.e., active coping, support coping, and politically oriented coping). Two of the coping resources considered are also associated with drinking patterns. Avoidant coping is associated with higher levels of past-month drinking and problematic drinking, whereas politically oriented coping is associated with less alcohol consumption and problematic drinking.

Formal mediation tests next reveal that the effects of economy-related stressors on the alcohol-related outcomes assessed are partly explained by variation in avoidant coping and politically oriented coping. Significant indirect effects are found for the pathways from economy-related stress to both past-month drinking and problematic drinking. Of the total effect of economy-related stress on past-month drinking (0.071), 0.047 is accounted for by the indirect effect of economy-related stress through avoidant coping and −0.024 is explained by the indirect influence of politically oriented coping. A similar pattern emerges with respect to the relationship between economy-related stressors and problematic drinking. The indirect effects of avoidant coping account for 0.046 of the total effect of economic stressors on problematic drinking (0.098) and politically oriented coping explains −0.032. Taken together, these findings indicate that both adaptive and maladaptive coping strategies come to bear on drinking patterns associated with economic strain. On the one hand, economic strain appears to be associated with drinking more and more problematically, in the extent to which it is associated with a tendency to engage in avoidant coping strategies. But, then, again, economic strain is also associated with greater politically oriented coping, which is protective against alcohol use and misuse.

**Figure 4:**
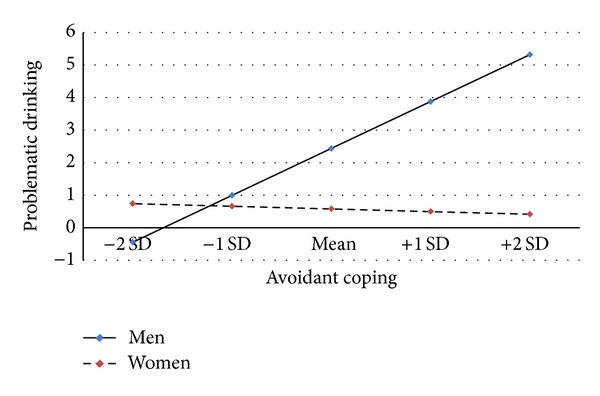
Gender contrasts in the effects of avoidant coping on problematic drinking. Notes: gender ∗avoidant coping (*β* = −0.319, s.e. = 0.021, and *P* < 0.001).

Moderation tests next determine whether the mediating effects of the coping strategies investigated vary by gender. No significant effects are observed for the pathway to past-month drinking patterns. However, the mediating effect of avoidant coping for the economy-related stressor problematic drinking association differs for men and women in this sample. This effect is displayed in Figure 4, which presents the predicted pattern of gender contrasts in the effects of avoidant coping on problematic drinking based on the mean, plus and minus two standard deviation values of avoidant coping (as displayed in Table 1). As shown in Figure 4, the mediating effects of avoidant coping differ for men and women because the relationship between avoidant coping and problematic drinking is significantly less strong for women compared to men (*β* = −0.319, s.e. = 0.021, and *P* < 0.001), net of the remaining variables. Thus, the observation that economic strain is associated with greater problematic drinking in the extent to which it is associated with avoidant coping strategies appears to be more pronounced among men than among women.

## 4. Discussion

The findings from this study support a broadened conceptualization of modes of coping in stress paradigm-oriented research to encompass politically oriented coping, in addition to modes of coping outside of the political realm which have predominated in studies of the coping-related moderators or mediators of the associations between social stressors and drinking outcomes. Moreover, given our focus on economic stressors deriving, at least in part, from the macrolevel social forces producing the Great Recession, we suggest that politically oriented coping is particularly salient as a mode of behavioral adaptation in relation to societally engendered stressors as opposed to stressors that are less affected by macrolevel social forces.

While this study specifically addressed deleterious drinking consequences of the recent Great Recession, social scientists have also delineated broader social-structural and political forces occurring over the last three decades which have led to pervasive job insecurity across all sectors of the US workforce and the erosion in the standard of living for most of the population [41–43]. These phenomena include globalization and the outsourcing of work, the downsizing of corporate entities, the shift from secure semiskilled industrial jobs which paid a living wage to low wage service sector jobs, an increase in contingent workers with lower pay, and lack of job security and fringe benefits. Thus, politically oriented coping encompassing the goal of changing governmental policies (e.g., rallying for changes in the tax structure influencing the distribution of wealth throughout society, fighting for government stimulus policies oriented toward job creation, or rallying support for raising the government-mandated minimum wage) may prove to be a more efficacious mode of coping with economic stressors and more protective against deleterious drinking outcomes compared to nonpolitically oriented active coping activities such as looking for a job when unemployed, especially if adequate numbers of jobs relative to demand do not exist and a large proportion of jobs that do exist pay less than a living wage.

Consistent with our gender-linked hypothesis, our data showed that males and females did not differ either in the use of politically oriented coping or in the extent to which politically oriented coping was protective in relation to drinking outcomes. However, our data showed that male but not female avoidant coping significantly predicted problem drinking. Thus, with regard to nonpolitically oriented coping with economic stressors, males clearly utilized a coping mode that was maladaptive. This finding might be seen as congruent with early male socialization patterns which have been viewed as fostering men's sense of self-importance rather than connectedness in social relationships [44] insofar as men's avoidance in dealing with economic problems may affect both themselves and others close to them. Alternatively, avoidant coping may predict problem drinking in men but not women to the extent to which males are socialized to drink more heavily than women.

Our findings should be viewed within the context of the methodological limitations of this study. The politically oriented coping measure which we developed for this study represents an initial attempt to operationalize this concept. However, it is limited to 4 items in contrast to the much longer nonpolitically oriented coping instrument and does not differentiate politically oriented coping tactics into discrete modes, similar to the measurement of nonpolitically oriented coping. Although factor analysis supported the inclusion of the indicators of politically oriented coping as a single measure, additional work on the concept of politically oriented coping would be useful, with a differentiation between alternative types of politically oriented coping. First, the differentiation between individual modes of political action (e.g., voting, signing petitions, and donating money) and collective political activities (participation and leadership roles in different types of political action groups) would be useful. Secondly, some types of collective coping could be viewed as relatively more adaptive versus maladaptive. For example, the Tea Party social movement was motivated by both economic and cultural concerns [45, 46]. However, one might differentiate between the Tea Party articulation of problem-focused social policies such as its belief in the need to diminish the size of government and racially oriented collective venting, such as screams of “kill him” by audience members in response to Sarah Palin's critique of Barak Obama at rallies during the 2008 presidential election [45].

Second, this study utilized cross-sectional data and, thus, likely provides only a snapshot of the complex processes linking economy-related stressors, coping strategies, and drinking outcomes. While our theoretical framework postulated that exposure to economic stressors coupled with particular modes of coping leads to problematic drinking, it is possible that problem drinking coupled with maladaptive coping might make one prone to experiencing economic stressors such as losing one's job. Moreover, we lack data regarding alcohol consumption prior to exposure to the stressors focused on in this study and the extent to which alcohol may have been used as a coping mechanism. Thus, further longitudinal studies are necessary to more clearly delineate the causal directions of the relationships between economic stressors, modes of coping, and drinking outcomes.

Third, the study methodology encompassed random-digit-dialing for recruiting the sample, thus only reaching individuals with landline telephone numbers. Consequently, individuals relying on cell phones only, along with households without access to any telephone, were not included in this study. This potential noncoverage error is a source of concern because comparisons of our data with the US population revealed that the sample underrepresented African Americans, Latinos, and younger (<age 40) and less-educated (high school or less) persons. However, our data were weighted to reflect the demographics of the overall population, and we compared weighted and unweighted estimates of each of our dependent variables to determine if nonresponse and/or noncoverage may have introduced serious bias into one or more of them. In each instance, we found that the weighted values of each measure fell well within 1 SD of the unweighted values, suggesting that the distributions of our key measures were not appreciably influenced by the underrepresentation of particular demographic groups.

## 5. Conclusions

Despite the noted limitations, this study extends prior research on the moderators and mediators of the social stressor-drinking outcome relationships to broaden notions of coping to include politically oriented coping. Future studies incorporating this mode of coping may more clearly elucidate the political dynamics involved in both the macrolevel production of social stressors and the deleterious alcohol-related consequences of these stressors. This line of research would also have implications for the treatment of alcohol-related problems. In particular, considerations of more adaptive modes of coping might go beyond recommending individual behaviors such as job seeking by unemployed individuals to also suggest politically oriented coping to collectively try to influence the social conditions such as unemployment levels that may give rise to the propensity to self-medicate distress through the use of alcohol.

## Figures and Tables

**Table 1:** Characteristics of study variables (*N* = 663).

Characteristics	Range	Mean	Standard deviation
Past-month drinking patterns	0–14	5.389	2.307
Problematic drinking	0–7	0.580	1.046
Economy-related stressors	0–48	13.553	9.863
Active coping	0–24	9.630	5.790
Support coping	0–12	2.808	2.767
Avoidant coping	0–25	5.702	4.769
Politically oriented coping	0–16	3.240	3.307
Gender (% female)	0,1	51.3	—
Age	19–91	54.838	14.713
Education (%)			
Less than high school	0,1	6.8	—
High school graduate	0,1	53.4	—
College graduate	0,1	16.8	—
Postcollege training	0,1	23.0	—
Race/ethnicity (%)			—
Non-Hispanic White	0,1	67.0	—
African American	0,1	12.2	—
Hispanic	0,1	13.9	—
Asian	0,1	4.4	—
Other	0,1	2.5	—

**Table 2:** Correlation matrix of drinking patterns, economic stressors, and coping strategies (*N* = 663).

	1	2	3	4	5	6	7	8
(1) Past-month drinking	1.0000							
(2) Problematic drinking	0.309∗∗∗	1.0000						
(3) Economy-related stressors	0.138∗∗∗	0.134∗∗∗	1.0000					
(4) Active coping	0.044	0.062	0.419∗∗∗	1.0000				
(5) Support coping	0.012	0.07	0.311∗∗∗	0.621∗∗∗	1.0000			
(6) Avoidant coping	0.115∗∗	0.129∗∗	0.473∗∗∗	0.529∗∗∗	0.501∗∗∗	1.0000		
(7) Politically oriented coping	−0.122∗∗	−0.118∗∗	0.230∗∗∗	0.129∗∗	0.115∗∗	0.088∗	1.0000	
(8) Gender (1 = female)	−0.182∗∗∗	−0.107∗	0.026	0.062	0.146∗∗∗	0.069	–0.002	1.0000

Note: ∗significant at 0.05; ∗∗significant at 0.01; ∗∗∗significant at 0.001. The Spearman correlation coefficients are presented for gender; for all other variables, the Pearson correlation coefficients are reported.

## References

[1] Cooper ML, Russell M, Skinner JB, Frone MR, Mudar P (1992). Stress and Alcohol Use: moderating Effects of Gender, Coping, and Alcohol Expectancies. *Journal of Abnormal Psychology*.

[2] Richman JA, Rospenda KM, Flaherty JA, Freels S (2001). Workplace harassment, active coping, and alcohol-related outcomes. *Journal of Substance Abuse*.

[3] Pearlin LI, Schooler C (1978). The structure of coping. *Journal of Health and Social Behavior*.

[4] Lazarus RS, Folkman S (1984). *Stress, Appraisal, and Coping*.

[5] Cooper ML, Frone MR, Russell M, Mudar P (1995). Drinking to regulate positive and negative emotions: a motivational model of alcohol use. *Journal of Personality and Social Psychology*.

[6] Skodol AE, Dohrenwend BP (1998). Personality and coping as stress-attenuating or amplifying factors. *Adversity, Stress, and Psychopathology*.

[7] Galea S, Galea S (2007). Integrative chapter: modifying macrosocial factors to improve population health. *Macrosocial Determinants of Population Health*.

[8] Link BG, Phelan J (1995). Social conditions as fundamental causes of disease. *Journal of Health and Social Behavior*.

[9] Richman JA, Cloninger L, Rospenda KM (2008). Macrolevel stressors, terrorism, and mental health outcomes: broadening the stress paradigm. *The American Journal of Public Health*.

[10] Turner RJ, Lloyd DA (1995). Lifetime traumas and mental health: the significance of cumulative adversity. *Journal of health and social behavior*.

[11] Treas J (2010). The great American recession: sociological insights on blame and pain. *Sociological Perspectives*.

[12] Richman JA, Rospenda KM, Johnson TP (2012). Drinking in the age of the great recession. *Journal of Addictive Diseases*.

[13] Gaby S, Caren N (2012). Occupy Online: How Cute Old Men and Malcolm X Recruited 400,000 US Users to OWS on Facebook. *Social Movement Studies*.

[14] Langman L (2013). Occupy: a new new social movement. *Current Sociology*.

[15] Flanagan C, Bundick M (2011). Civic engagement and psychological well-being in college students. *Liberal Education*.

[16] Zimmerman MA, Rappaport J (1988). Citizen participation, perceived control, and psychological empowerment. *The American Journal of Community Psychology*.

[17] Carver CS (1997). You want to measure coping but your protocol’s too long: consider the brief COPE. *International Journal of Behavioral Medicine*.

[18] Kaplan HB, Liu X, Stryker S, Owens T, White RW (2000). Social movements as collective coping with spoiled personal identities: intimations from a panel study of changes in the life course between adolescence and adulthood. *Self, Identity, and Social Movements*.

[19] Thoits PA (2006). Personal agency in the stress process. *Journal of Health and Social Behavior*.

[20] Billings AG, Moos RH (1981). The role of coping responses and social resources in attenuating the stress of life events. *Journal of Behavioral Medicine*.

[21] Felsten G (1998). Gender and coping: Use of distinct strategies and associations with stress and depression. *Anxiety, Stress and Coping*.

[22] Matud MP (2004). Gender differences in stress and coping styles. *Personality and Individual Differences*.

[23] Verba S, Burns N, Schlozman KL (1997). Knowing and caring about politics: gender and political engagement. *The Journal of Politics*.

[24] Coffé H, Bolzendahl C (2010). Same game, different rules? Gender differences in political participation. *Sex Roles*.

[25] Lazarus RS (1999). *Stress and Coping: A New Synthesis*.

[26] Lavrakas PJ (1993). *Telephone Survey Methods: Sampling, Selection, and Supervision*.

[27] American Association for Public Opinion Research (2011). *Standard Definitions: Final Dispositions of Case Codes and Outcome Rates for Surveys*.

[28] Centers for Disease Control and Prevention (2010). *Behavioral Risk Factor Surveillance System Survey Data*.

[29] Cahalan D, Cisin IH, Crossley HM (1969). *American Drinking Practices: A National Study of Drinking Behavior and Attitudes*.

[30] Fitzgerald JL, Mulford HA (1987). Self-report validity issues. *Journal of Studies on Alcohol*.

[31] Pokorny AD, Miller BA, Kaplan HB (1972). The brief MAST: a shortened version of the Michigan Alcoholism Screening Test. *The American Journal of Psychiatry*.

[32] Maisto SA, Connors GJ, Allen JP (1995). Contrasting self-report screens for alcohol problems: a review. *Alcoholism, Clinical and Experimental Research*.

[33] Brown RL, Richman JA (2012). Sex differences in mediating and moderating processes linking economic stressors, psychological distress, and drinking. *Journal of Studies on Alcohol and Drugs*.

[34] Farley T, Galves A, Dickinson LM, de Jesus Diaz Perez M (2005). Stress, coping, and health: a comparison of Mexican immigrants, Mexican-Americans, and non-Hispanic Whites. *Journal of Immigrant Health*.

[35] Feaster DJ, Szapocznik J, Smith L, Samuels D, Dadson MJ, Antoni M (2000). The influence of stress, family problems, support, and coping on psychological distress among HIV+ and HIV- urban low-income African American mothers,. *Technical Report*.

[36] Greening L, Stoppelbein L (2007). Brief report: pediatric cancer, parental coping style, and risk for depressive, posttraumatic stress, and anxiety symptoms. *Journal of Pediatric Psychology*.

[37] Muthén LK, Muthén BO (2010). *Mplus User's Guide*.

[38] MacKinnon DP, Warsi G, Dwyer JH (1995). A simulation study of mediated effect measures. *Multivariate Behavioral Research*.

[39] Richman JA, Shinsako SA, Rospenda KM, Flaherty JA, Freels S (2002). Workplace harassment/abuse and alcohol-related outcomes: The mediating role of psychological distress. *Journal of Studies on Alcohol*.

[40] Hu L-T, Bentler PM (1999). Cutoff criteria for fit indexes in covariance structure analysis: conventional criteria versus new alternatives. *Structural Equation Modeling*.

[41] Bluestone B, Harrison B (1982). *The Deindustrialization of America: Plant Closings, Community Abandonment, and the Dismantling of Basic Industry*.

[42] Kalleberg AL (2011). *Good Jobs, Bad Jobs: The Rise of Polarized and Precarious Employment Systems in the United 
States, 1970s to 2000s*.

[43] Stiglitz JE (2012). *The Price of Inequality: How today’s Divided Society Endangers our Future*.

[44] Rosenfield S, Vertefuille J, Mcalpine DD (2000). Gender stratification and mental health: an exploration of dimensions of the self. *Social Psychology Quarterly*.

[45] Langman L (2012). Cycles of contention: the rise and fall of the tea party. *Critical Sociology*.

[46] Skocpol T, Williamson V (2012). *The Tea Party and the Remaking of Republican Conservatism*.

